# Incidence of Postoperative Diabetes Mellitus After Roux-en-Y Reconstruction for Gastric Cancer: Retrospective Single-Center Cohort Study

**DOI:** 10.2196/56405

**Published:** 2024-08-14

**Authors:** Tatsuki Onishi

**Affiliations:** 1 Data Science and AI Innovation Research Promotion Center, Shiga University, 1 Chome-1-1 Bamba, Hikone, Shiga, 522-0069, Japan, 81 749 27 1030; 2 Department of Anesthesia, Juntendo University Shizuoka Hospital, Izunokuni, Shizuoka, Japan; 3 Department of Anesthesia, Kyowa Hospital, Kyoto, Japan

**Keywords:** diabetes mellitus, gastrectomy, gastric cancer surgery, glucose metabolism, postoperative diabetes onset, surgery outcomes

## Abstract

**Background:**

Sleeve gastrectomy is an effective surgical option for morbid obesity, and it improves glucose homeostasis. In patients with gastric cancer and type 2 diabetes mellitus (DM), gastrectomy, including total gastrectomy, is beneficial for glycemic control.

**Objective:**

This study aims to clarify the effects of gastrectomy and different reconstructive techniques on the incidence of postoperative DM in patients with gastric cancer.

**Methods:**

This retrospective, single-center, cohort study included 715 patients without DM who underwent total gastrectomy at the Tokyo Metropolitan Bokutoh Hospital between August 2005 and March 2019. Patients underwent reconstruction by Roux-en-Y (RY) gastric bypass or other surgical techniques (OT), with DM onset determined by hemoglobin A_1c_ levels or medical records. Analyses included 2-sample, 2-tailed *t* tests; *χ*^2^ tests; and the Kaplan-Meier method with log-rank tests to compare the onset curves between the RY and OT groups, along with additional curves stratified by sex. A Swimmer plot for censoring and new-onset DM was implemented.

**Results:**

Stratified data analysis compared the RY and OT reconstruction methods. The hazard ratio was 1.52 (95% CI 1.06-2.18; *P*=.02), which indicated a statistically significant difference in the incidence of new-onset diabetes between the RY and OT groups in patients with gastric cancer. The hazard ratio after propensity score matching was 1.42 (95% CI 1.09-1.86; *P*=.009).

**Conclusions:**

This first-of-its-kind study provides insight into how different methods of gastric reconstruction affect postoperative diabetes. The results suggest significant differences in new-onset DM after surgery based on the reconstruction method. This research highlights the need for careful surgical planning to consider potential postoperative DM, particularly in patients with a family history of DM. Future studies should investigate the role of gut microbiota and other reconstructive techniques, such as laparoscopic jejunal interposition, in developing postoperative DM.

## Introduction

Gastrectomy, particularly sleeve gastrectomy (SG), has been shown to be an effective surgical option for morbid obesity due to its low complication rates and significant weight loss results [1-5]. SG results in alteration of the appetite through the regulation of gut hormones, resulting in decreased hunger and increased satiety [6]. SG also improves glucose homeostasis through resulting changes in gut hormone levels [7]. Specifically, laparoscopic SG results in significant improvement in glucose metabolism in patients who are morbidly obese and has been found to stop the development of diabetes at a high rate [8]. SG has been shown to improve blood glucose independently of weight loss by restoring hepatic insulin sensitivity [9]. However, the effects of gastrectomy on patients who are not obese with type 2 diabetes mellitus (DM) are less clear, with some studies suggesting that gastrectomy may improve diabetic status [10].

In patients with gastric cancer diagnosed with type 2 DM, gastrectomy has been found to have a positive impact on their glycemic control. Improvements in glycemic control, or even diabetes remission, have been reported after gastrectomy [10-15]. The extent of the gastrectomy, duration of diabetes, and method of reconstruction have been identified as important factors influencing the improvements in glycemic control [10-14]. Although the mechanisms underlying these effects are not fully understood, oncometabolic surgeries, including gastrectomy, have been suggested as a potential treatment for type 2 DM in patients with gastric cancer [16].

Studies have shown that total gastrectomy (TG) is associated with improved glucose metabolism in patients with gastric cancer, resulting in a lower rate of newly diagnosed diabetes after surgery [17]. However, the effects of gastrectomy on glucose metabolism in patients with and without diabetes have been inconsistent, with some studies reporting significant reductions in fasting blood glucose levels after gastrectomy [18]. Furthermore, SG has been associated with significant reductions in hemoglobin A_1c_ levels in patients without diabetes, suggesting its possible role in the prevention of type 2 DM [19].

In terms of reconstruction after partial gastrectomy in patients with gastric cancer, both Roux-en-Y (RY) and Billroth II reconstructions have been considered acceptable options [20]. RY reconstruction is often preferred for patients with gastric cancer, given that this procedure can lead to decreased reflux gastritis and esophagitis, decreased probability of cancer recurrence, and decreased incidence of surgical complications [21]. RY reconstruction has also been found to be as effective as other methods with respect to nutritional status and postoperative outcomes [22]. In comparison to Billroth II reconstruction, RY has been shown to have similar postoperative complications and better long-term outcomes [23]. Furthermore, RY reconstruction without cutting has been the preferred method in cases of gastritis, bile reflux, and gastric residuals [24].

Various studies have examined the impact of different reconstructive procedures on postoperative complications in patients with gastric cancer. It has been found that long-limb RY bypass reconstruction could lead to improved glycemic control [25], and it has been observed that preexisting DM is associated with postoperative complications [10,26]. Several studies further support the benefits of RY reconstruction, with some indicating it to be more effective than Billroth II reconstruction [27,28]. Additionally, significant improvements in DM control have been associated with RY reconstruction [25,28,29].

Given these, the aim of this study was to investigate the incidence of new-onset DM in patients with gastric cancer after surgery and how this incidence varies with different types of surgical reconstruction, namely, the RY procedure and other alternative reconstruction techniques. While studies have investigated how surgical treatment for gastric cancer affects existing DM [25,28,29], none have investigated the development of new-onset DM in patients without DM; to the best of our knowledge, this is the very first study to do so. Findings from this study could contribute valuable insights into the postoperative outcomes associated with different gastric reconstruction techniques. Such insights are vital for guiding clinical decisions and optimizing patient care, particularly in the context of mitigating the risk of developing DM after gastric surgery. Moreover, findings from this study are expected to have significant implications for both clinical practice and future research in the field of gastric surgery and DM prevention.

## Methods

### Ethical Considerations

The study was approved by the Tokyo Metropolitan Bokutoh Hospital’s ethics committee (30-110) and conducted in accordance with the Declaration of Helsinki. Individual informed consent was waived because the data were deidentified and not trackable. No compensation was given.

### Study Participants

The study design was a retrospective, single-center, cohort study. A total of 715 patients who underwent TG as the definitive procedure and as a standby procedure at the Tokyo Metropolitan Bokutoh Hospital between August 2005 and March 2019 and were not diagnosed with DM at the time of surgery were included in the study. Whether the patients would undergo reconstruction through RY gastric bypass or other surgical techniques (OT) was chosen based on the preference of the surgeons, and the patients were grouped accordingly. The definite onset of diabetes in the patients was considered based on previous electronic medical records or when their hemoglobin A_1c_ value was equal to or greater than 6.5 based on laboratory testing. The competing outcome was death. After a meticulous data curation process using Python (version 3.10; Python Software Foundation) that corrects for missing values and ensures appropriate data types, we obtained a dataset that was optimized for analysis and free of common data inconsistencies.

### Statistical Analysis

Basic statistical measures such as the mean, median, and SD were computed. Two-sample, 2-tailed *t* tests and chi-square tests were used to assess the difference in demographic characteristics between the 2 groups. In addition, the Kaplan-Meier method was used to estimate the onset function delineating the interval between the TG and the subsequent emergence of new-onset diabetes postoperatively and was augmented with log-rank tests to help compare the onset curves between the RY and OT groups, along with additional curvesstratified by sex, and the same analysis was carried out after propensity score matching. A Swimmer plot for censoring and new-onset DM was implemented. The abovementioned analyses were conducted using Python (version 3.10).

## Results

The characteristics of the patients included in the study at the time of the surgery are shown in Table 1. Of the 715 patients who had a gastrectomy, 489 (68.4%) underwent RY reconstruction.

**Table 1.** Demographics of study population.

Characteristics		Missing data (N=715), n (%)	OT^a^ (n=226)	RY^b^ (n=489)	*P* value
Cases (N=715), n (%)		—^c^	226 (31.6)	489 (68.4)	—
Age (years), mean (SD)		—	70.0 (10.4)	68.1 (10.6)	.03^d^
Male sex, n (%)		—	139 (61.5)	363 (74.2)	.001^e^
Height (cm), mean (SD)		—	157.9 (10.8)	160.6 (8.6)	.001^d^
Weight (kg), mean (SD)		—	56.9 (14.8)	57.1 (11.1)	.87^d^
BMI (kg/m^2^), mean (SD)		—	24.6 (32.2)	22.1 (3.4)	.25^d^
**ASA-PS^f^, n (%)**		—			.03^e^
	1		21 (9.3)	27 (5.5)	
	2		167 (73.9)	384 (78.5)	
	3		34 (15)	77 (15.7)	
	4		4 (1.8)	1 (0.2)	
Total intravenous anesthesia, n (%)		—	6 (2.7)	43 (8.8)	.004^e^
Nerve block, n (%)		—	210 (92.9)	462 (94.5)	.52^e^
Bleeding (mL), mean (SD)		—	362.5 (301.7)	582.8 (639.1)	<.001^d^
Blood transfusion (mL), mean (SD)		—	23.8 (111.9)	108.6 (373.3)	<.001^d^
Urine (mL), mean (SD)		—	364.4 (319.7)	383.0 (321.1)	.47^d^
Infusion (mL), mean (SD)		—	2185.4 (697.0)	2545.5 (929.5)	<.001^d^
Operating room time (min), mean (SD)		—	298.4 (74.4)	317.5 (76.8)	.002^d^
Anesthesia time (mL), mean (SD)		—	275.5 (75.0)	295.7 (76.3)	.001^d^
Operation time (mL), mean (SD)		—	225.2 (69.6)	244.5 (71.8)	.001^d^
**T^g^ (OT: n=126, RY: n=252), n (%)**		337 (47.1)			<.001^e^
	1		56 (44.4)	64 (25.4)	
	2		46 (36.5)	81 (32.1)	
	3		20 (15.9)	88 (34.9)	
	4		4 (3.2)	17 (6.7)	
	0		0 (0)	2 (0.8)	
**M^h^ (OT: n=126, RY: n=251), n (%)**		338 (47.3)			.35^e^
	0		123 (97.6)	248 (98.8)	
	1		3 (2.4)	2 (0.8)	
	3		0 (0)	1 (0.4)	
**N^i^ (OT: n=125, RY: n=249), n (%)**		341 (47.7)			<.001^e^
	0		76 (60.8)	94 (37.8)	
	1		29 (23.2)	71 (28.5)	
	2		19 (15.2)	63 (25.3)	
	3		1 (0.8)	21 (8.4)	
**D^j^ (OT: n=120, RY: n=234), n (%)**		361 (50.5)			.002^e^
	0		2 (1.7)	5 (2.1)	
	1		72 (60)	88 (37.4)	
	2		46 (38.3)	139 (59.1)	
	3		0 (0)	2 (0.9)	

a OT: other surgical techniques.

b RY: Roux-en-Y reconstruction.

c Not applicable.

d 2-sample, 2-tailed *t* test.

e *χ*2 test.

f ASA-PS: American Society of Anesthesiologists Physical Status.

g T: tumor (TNM staging).

h M: metastasis (TNM staging).

i N: node (TNM staging).

j D: dissection.

**Figure 1.**
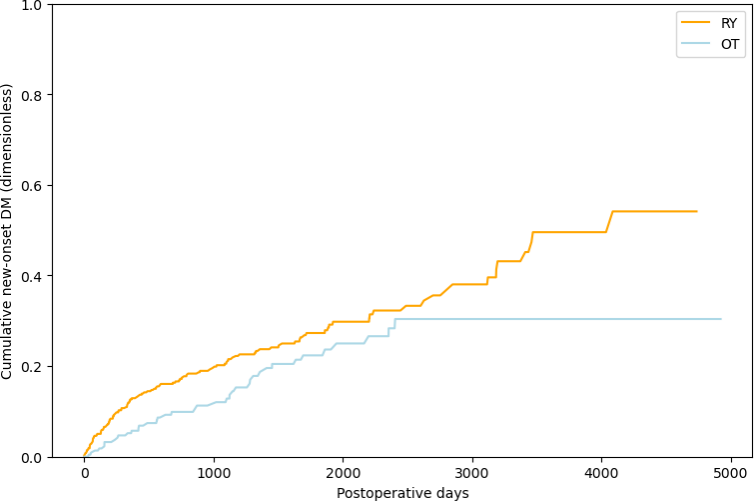
Kaplan-Meier curve of new-onset DM in the RY and OT groups. DM: diabetes mellitus; OT: other surgical techniques; RY: Roux-en-Y.

The Kaplan-Meier curve of new-onset DM in the RY and OT groups is shown in Figure 1. Granular comparison of the incidence rates of postoperative diabetes associated with these distinct reconstructive procedures was made. The rate of diabetes onset was inferred from the slope of these curves, with a steeper decline indicating a higher incidence within the respective group.

A log-rank test revealed that the hazard ratio was 1.52 (95% CI 1.06-2.18), and the resultant *P* value from this log-rank test was .02, which denotes a statistically significant difference in the incidence of new-onset diabetes after surgery between patients with gastric cancer who underwent RY reconstruction versus OT. These findings indicate a difference in the incidence of postoperative diabetes based on the type of gastric reconstruction method used (Figure 1).

**Figure 2.**
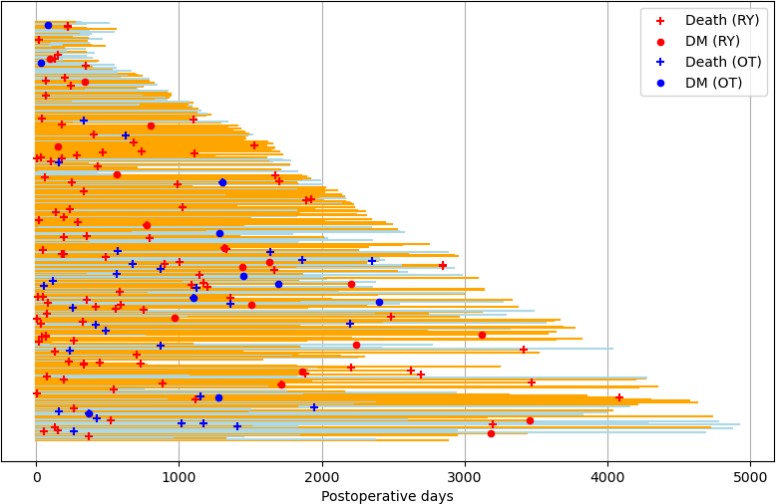
Swimmer plot of new-onset DM and death. DM: diabetes mellitus; OT: other surgical techniques; RY: Roux-en-Y.

A Swimmer plot was then produced (Figure 2). In the Swimmer plot, orange lines represent RY cases, blue lines represent OT cases, a cross indicates censoring due to death, and a circle represent censoring due to new-onset DM. The last DM onset in the OT group was at approximately 2400 days, which explains the linear part of the Kaplan-Meier curve.

**Figure 3.**
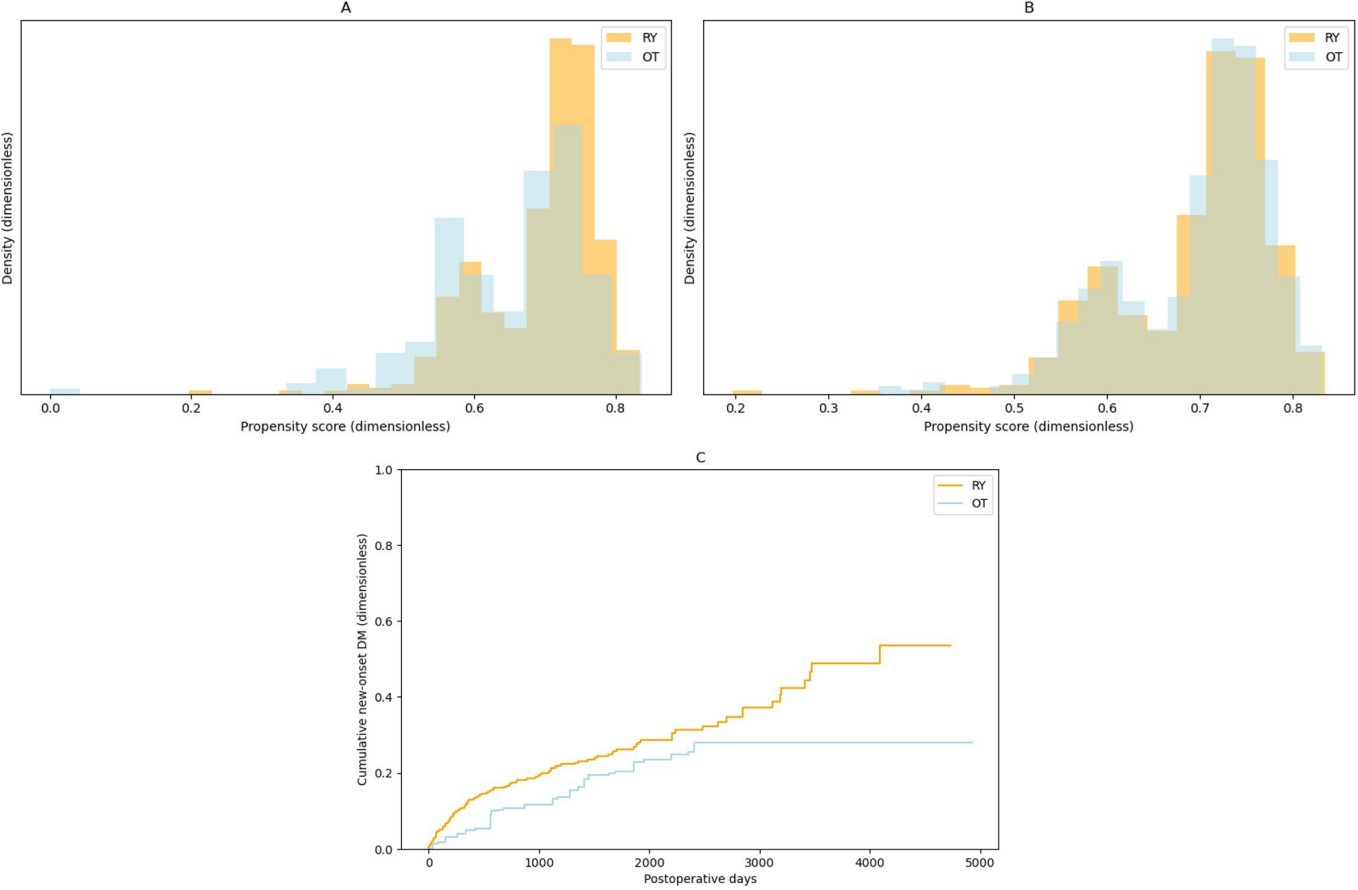
Density histogram (A) before and (B) after propensity score matching, and (C) Kaplan-Meier curve of new-onset DM after propensity score matching. DM: diabetes mellitus; OT: other surgical techniques; RY: Roux-en-Y.

Propensity score matching was conducted according to the use of laparoscopy, age, sex, and BMI. After propensity score matching, the Kaplan-Meier onset curve showed a hazard ratio of 1.42 (95% CI 1.09-1.86), and the resultant *P* value was .009 (Figure 3). This means that the results are robust even when accounting for unknown confounding and that RY cases have more postoperative DM than OT cases.

**Figure 4.**
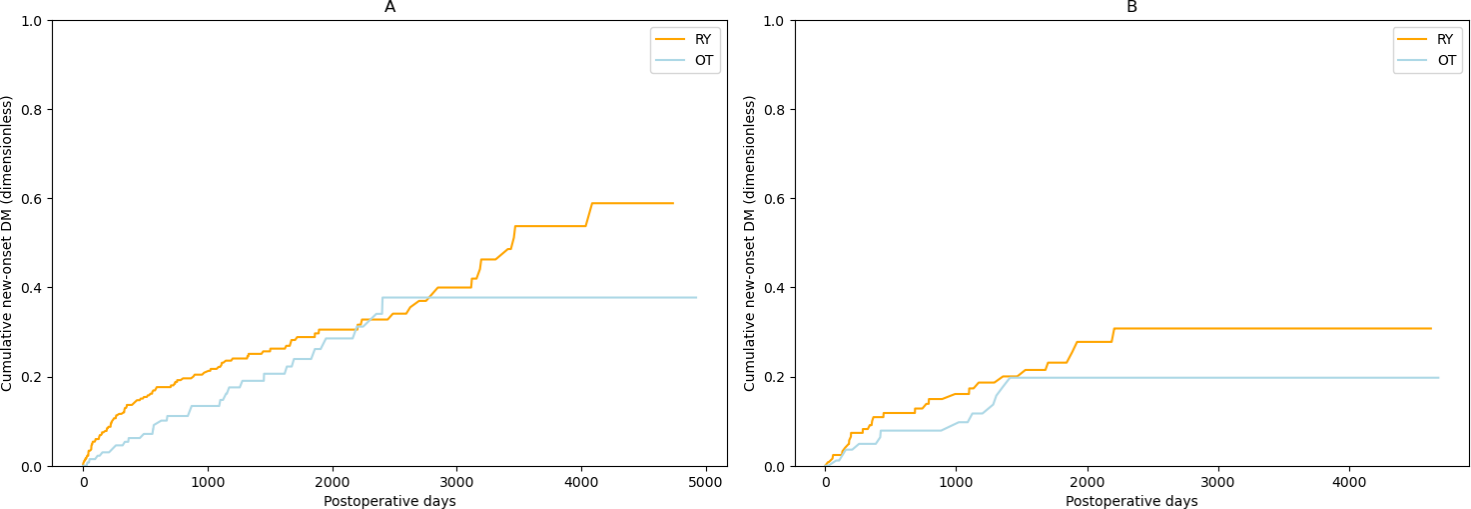
Kaplan-Meier curve of new-onset DM stratified by sex: (A) male and (B) female. DM: diabetes mellitus; OT: other surgical techniques; RY: Roux-en-Y.

A Kaplan-Meier curve stratified by sex was also generated (Figure 4). In this Kaplan-Meier curve, there was no significant difference in the development of postoperative DM between the RY and OT groups for both male and female patients (*P*=.12 and *P*=.24, respectively).

## Discussion

### Principal Findings

This study showed that patients who underwent RY reconstruction had more postoperative DM than those who underwent OT. This study is the first to provide insights into how different methods of gastric reconstruction might affect the risk of developing postoperative DM.

### Comparison to Prior Work

Although there were works reporting the influence of preexisting DM after gastrectomy [25,28,29], there was no work regarding new-onset DM after gastrectomy with distinct surgical reconstruction techniques. Some studies mentioned a change in microbiota after gastrectomy [30,31]. Specific changes in the gut microbiota after surgery include increased species richness, decreased butyrate-producing bacteria, and enrichment of certain symbiotic bacteria [32]. The abundance of specific gut bacterial genera has been found to correlate with the population of peripheral immune cells [32-34].

### Strengths

A thorough approach to data preprocessing and the use of robust statistical methods will ensure the reliability and validity of these findings in the wider context of gastric surgery and DM research. Our study is important for understanding the temporal dynamics of DM development after gastric surgery and has significant implications for surgical planning and patient management to prevent postoperative DM, especially in patients with a strong family history of DM.

### Limitations

Deaths for any reason was 14.2% (32/226) in the OT group and 21.7% (106/489) in the RY group (*P*=.02 by *χ*^2^ test), which may have influenced the interpretation of the results. This study did not include an assessment of other determinants that could potentially influence the development of DM, including lifestyle choices and genetic predisposition. It is plausible that there may be a difference in the intrinsic characteristics of DM in patients who present with diabetic symptoms prior to undergoing surgery for gastric cancer, as opposed to those in whom the onset of gastric malignancy precedes the development of DM. Such considerations were beyond the scope of analysis within the parameters of this study.

### Future Directions

First, laparoscopic jejunal interposition reconstruction (LJIP), a surgical technique in which a pouch is created in the jejunum to reconstruct the upper gastrointestinal tract, may be appropriate for patients with impaired glucose tolerance [35]. Studies have shown that LJIP leads to better postoperative outcomes, including improved quality of life and nutritional status, compared with other reconstruction methods [35-38]. Future study should include LJIP as a reconstructive method.

Second, it was not possible to study the gut microbiota. With access to a suitable dataset, we would like to investigate the association between gut microbiota and the development of new-onset DM after gastrectomy.

Third, our study could be improved by comparison with a population that has not undergone TG as a control.

## Abbreviations

DM: diabetes mellitus
LJIP: laparoscopic jejunal interposition reconstruction
OT: other surgical techniques
RY: Roux-en-Y
SG: sleeve gastrectomy
TG: total gastrectomy
